# A Multicenter, Randomized, Parallel-Group Trial Assessing Compliance, Tolerability, Safety, and Efficacy to Treatment with Grass Allergy Tablets in 261 Patients with Grass Pollen Rhinoconjunctivitis

**DOI:** 10.1155/2012/673502

**Published:** 2011-11-09

**Authors:** Roberta Alesina, Massimo Milani, Silvia Pecora

**Affiliations:** ^1^Divisione di Pneumologia, Ospedale San Matteo, Pavia, Italy; ^2^ALK-Abellò Italy Medical Department, Via Settembrini 29, 20020 Lainate, Italy

## Abstract

*Background*. Allergen-specific sublingual immunotherapy (SLIT) is considered a causal treatment of respiratory allergies. Compliance to the SLIT is an important aspect for a positive clinical outcome. *Study Aim*. To evaluate if compliance with grass Allergy Immunotherapy Tablet (AIT) can be increased by providing an electronic compliance device (CED) (Memozax; a tablet-container with a programmable daily acoustic alarm). *Patients and Methods*. 261 patients with grass allergy were enrolled and randomized (1 : 1) to 1-year treatment with AIT (Grazax) using a CED (group A; *n* = 122) or without (Group B, *n* = 139). Compliance was measured through tablet count at each visit. *Results*. The 12-month compliance, mean (SD), in group A was 83% (21) and 83% (24) in group B. A total of 81% of patients reported a significant clinical improvement of symptoms after treatment in comparison with the previous year. No severe adverse reactions were observed in the study. *Conclusion*. Compliance to the treatment with AIT administered for 12 consecutive months is in general good. The use of CED is not associated with a greater compliance. AIT treatment was associated with a significant clinical improvement in >80% of patients with a good tolerability and safety profile.

## 1. Introduction

Allergic rhinoconjunctivitis represents a global health problem affecting 10 to 25% of the population [1]. Allergy to grass pollen is one of the most common inhalant allergies in the western world. In an unselected “healthy” population it has been found that 8 to 21% of children and 13% of adults are sensitized to grass pollen [2]. In selected populations of allergic subjects 44% are allergic to grass pollen [3]. Allergic rhinoconjunctivitis has been identified as one of the main reasons for visits to primary care clinics, and although usually not regarded as a severe disease it significantly limits the social life of the subject and affects school learning performance and work productivity [4]. Allergen-specific immunotherapy is the practice of administering to allergic patients increasing amount of allergen in order to obtain hyposensitization [5]. Allergen-specific immunotherapy is considered the only causal treatment of allergic diseases such as allergic rhinitis asthma and insect venom allergy. The purpose of allergen-specific immunotherapy is to expose the patient to the allergen that causes the allergic symptoms, in order to increase the tolerance to this allergen and reduce symptoms [6]. Mechanisms of action of allergen-specific immunotherapy are not so far clearly identified; however, data are available that allergen-specific immunotherapy can induce an increased production of allergen-specific IgG4 and IL-10 [7]. Alternative mechanisms include immune deviation in favour of TH1 responses and apoptosis and/or anergy of antigen-specific T cells. Allergen-specific immunotherapy exerts its beneficial effects over long periods (i.e., weeks or months), and 3-4 years of treatment are required to obtain a favourable clinical and immunological response [8]. The treatment with allergen-specific immunotherapy is currently administered as subcutaneous (s.c.) injections by specialists and reduces the allergic symptoms considerably [9]. The “standard” allergy vaccination program requires an updosing period followed by a maintenance period of 3–5 years [10]. This implies that only a fraction of allergic subjects are actually offered allergy vaccination despite the fact that allergen-specific immunotherapy is the only treatment modality that changes the natural cause of the allergic disease and thereby prevents its exacerbation. The treatment modality is well known as clinical application of *Phleum pratense *for s.c. administration has been carried out during the past 30 years in several European countries [11]. Allergen-specific sublingual immunotherapy (SLIT) has gained wide acceptance in many European countries and has raised the level of interest in immunotherapy among practicing allergists and primary care physicians. Large pivotal double-blind, placebo-controlled, randomized clinical trials have confirmed the efficacy and safety of SLIT. Allergen-specific sublingual immunotherapy with grass products has also had a widespread application—especially in Southern Europe throughout the past 30 years [12]. The general recommendation today is to apply a higher than the accumulated dosage applied subcutaneously. Grass allergy immunotherapy tablet (AIT) (*Grazax;* ALK Denmark) has been developed for allergen-specific immunotherapy [13]. Grazax is formulated as an orodispersible tablet for sublingual use and contains a standardised allergen extract derived from extraction and purification of the source material, *Phleum pratense *Timothy grass pollen [14]. To obtain an optimal therapeutic response with immunotherapy requires patients to be compliant with the recommendations given by the physicians. Maximal compliance can improve the patient's condition and also result in a reduction in drug costs [15]. Conversely, poor compliance may result in the physicians adding in more medications to treat the patient's condition, which may make the problem worse. Compliance to allergen-specific immunotherapy could be negatively influenced by several factors such as: duration of treatment, side effects, especially in the initiation phase, and need to take medication also outside the pollen season period when the patient in general does not have any symptoms. Specific allergen SLIT is a long-lasting home treatment that is directly managed by patients and parents. Therefore, as allergen-specific SLIT is self-managed at home without direct supervision, adequate compliance with this administration route is important.

## 2. Study Aim

The primary objective of the trial was to evaluate if compliance of once daily dosing with grass AIT in adult subjects with grass-pollen-induced allergic rhinoconjunctivitis could be increased by providing patients with compliance device (Memozax) (Figure 2) given from the beginning of immunotherapy in comparison with patients without the Memozax. Secondary endpoints of the trial were to evaluate safety and tolerability of grass AIT treatment and finally to evaluate the tolerability of the first dose intake of AIT and to evaluate after 48-week treatment with grass allergy tablet tablets the impact on symptom score and patient's acceptance in comparison with previous pollen seasons.

## 3. Patients and Methods

### 3.1. Study Design

This was a 23-centre, single-dose, randomized parallel-group, open-label, controlled trial.

### 3.2. Patients Selection

A total of 240 subjects were planned for enrolment. Enrolled patients were adult (>18 years), men or women, suffering from mild or moderate/severe grass-pollen-induced allergic rhinoconjunctivitis. A total of 261 patients were screened, enrolled, and randomized to 48-week treatment with AIT using the compliance aid device (Memozax) or 48-week treatment with AIT without the compliance device. The screening phase lasted 1 week. Therefore the total study duration was 49 weeks. All enrolled patients were treated with one tablet of AIT daily for 48 weeks.

### 3.3. Inclusion and Exclusion Criteria

Subjects were selected from the outpatient population of allergy clinics in Italy. Subject selection was based on the following criteria. Inclusion criteria were as follows: subjects, men and women >18 years of age and <65 years; suffering from mild or moderate/severe grass-pollen-induced rhinoconjunctivitis (according to ARIA Guidelines [16]) and with a positive SPT for *Phleum pratense* extract (≥3 mm); every patient should give a written informed consent to participate in the trial. Exclusion criteria at randomization were as follows: current symptoms of, or treatment for, upper respiratory tract infection, acute sinusitis, acute otitis media, or other relevant infectious processes; history of emergency visit or admission for asthma in the previous 12 months; use of an investigational drug within 30 days prior to screening; previous treatment by immunotherapy with grass pollen allergen; previous treatment by immunotherapy with other allergen than grass pollen allergen; within the previous 5 years; history of anaphylaxis, including anaphylactic food allergy, bee venom anaphylaxis, exercise anaphylaxis, or drug-induced anaphylaxis; or history of angioedema.

### 3.4. Therapeutic Regimen

The treatment used was Grazax oral lyophilisate 75,000 SQ-T tablets (*Phleum pratense* grass pollen allergen extract). The daily dose was one tablet, which should preferably be taken in the morning. The tablet was placed under the tongue and swallowing should be avoided for one minute. Eating and drinking was not allowed within five minutes after trial medication intake. The same drug taking instructions were given to both study groups (randomized to Memozax or not). Concomitant medications were all medications (including rhinoconjunctivitis medications and asthma treatments) being continued by a subject on entry to the trial and all medications given in addition to the treatment during the trial. All concomitant medications should have been documented in the CRF (trade name as appropriate). Further, each change in concomitant treatment (e.g., new treatment, discontinuation of treatment, and change in dosage/routine) during the trial must be documented in the same way. At each visit, the investigator asked the subject about concomitant medications. Any concomitant medication was recorded in the subject's notes as source data documentation and in the CRF. The investigators were instructed to store the drug in an appropriate, secure area (e.g., locked cabinet) and to store it according to the conditions specified on the labels. The investigator should have to maintain an accurate record of the shipment and dispensing of trial medication in a drug accountability log, a copy of which was given to ALK-Abelló at the end of the trial. An accurate record of the date and amount of trial medication dispensed to each subject was available for inspection at any time. The first dose was taken in the clinic and the subject stayed in the clinic for 60 minutes for observation. The first dose tolerability was recorded in the CRF. The following doses were taken at home. The experimental drug was supplied in blister cards containing 10 tablets each. The blister cards were packed in visit specific boxes.

### 3.5. Randomization Procedures

Between October 2007 and February 2008 a total of 240 subjects were planned to receive grass AIT as an oral lyophilisate once daily. The subjects were randomized (1 : 1) using a randomization list with half the subjects planned to receive the compliance device and half not to receive the device. Subjects were identified by ascending 2-digit randomization numbers, plus 2-digit referring to the centre and entered in the Case Report Form. When a subject was randomized in the trial he/she had to be assigned the lowest available randomization number for that centre. The randomization number was a 5-digit number where the two first digits gave a center code. Grass allergen tablet treatment was provided at the screening/randomization visit together with the Memozax, according to the randomization list. In all enrolled patients treatment started at least 3 months before the pollen season of 2008.

### 3.6. Compliance Evaluation

The primary outcome of the study was the evaluation and comparison of compliance in the two groups (with Memozax and without Memozax) evaluated with pill count at visits 3 (week 2), 4 (week 24), and 5 (week 48) calculated in the following manner: number of pill effectively taken/number of pill to be taken ×100. Subjects were instructed to return all residual and unused trial medications and all empty packaging at every visit. Compliance was assessed by tablet counts.

### 3.7. Secondary Endpoints of the Trial

Other efficacy assessment was to evaluate after 48 weeks of treatment with Grazax tablets the impact on quality of life, symptom score, and patient's acceptance in comparison with previous pollen seasons. This was evaluated, globally, through a 10 cm VAS scale (0 = big improvement, 5 = not improvement, and 10 = worsening of symptoms). The safety assessments included recording of all adverse events (AEs) and serious adverse events (SAEs) findings from physical examinations and vital signs.

### 3.8. Conduction of the Trial

This trial was conducted in compliance with the principles of *Good Clinical Practice*. The trial was monitored according to Sponsor Company standard operating procedures for the monitoring of clinical studies and other trial-specific procedures. The trial was monitored by the sponsor or its delegate by means of on-site visits, telephone calls, and regular inspection of the CRFs with sufficient frequency (every 8–12 weeks) to verify the following: subject enrolment; compliance with the protocol; the completeness and accuracy of data entered in the CRFs by verification against original source documents; compliance in the use of IMP; drug accountability; recording of adverse events.

### 3.9. Statistical Methods and Sample Size Calculation

All statistical analyses were carried out by SPSS statistical Package software. The following analysis set was defined in the protocol:* full-analysis set (FAS), *this consists of all subjects randomized following the intent-to-treat (ITT) ICH principle. The FAS was the only analysis set. A scientific publication has shown that compliance to SLIT treatment >90% without any device system was registered in 75% of treated patients. In this study it was hypothised that the group of patients with the Memozax device should have a better compliance (a relative increase of 15% or more) in comparison with the group without the aid device (86% of patients with a compliance of 90% or more in the Memozax group versus a 75% in the group without the Memozax). A minimum of 120 subjects per group, with an alfa error of 0.05 and a power of 80%, therefore should be enrolled in the trial. Actually a total of 261 patients were enrolled in this trial. The comparison between the two groups was analyzed using the ANOVA test. The comparison of percentage of patients with a compliance <90% and >90% between the two groups was performed with the Fisher exact test.

## 4. Results

**Figure 1:**
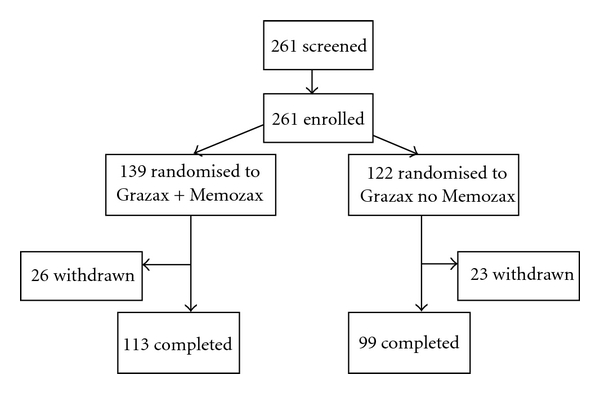
GT17 study flow.

**Figure 2:**
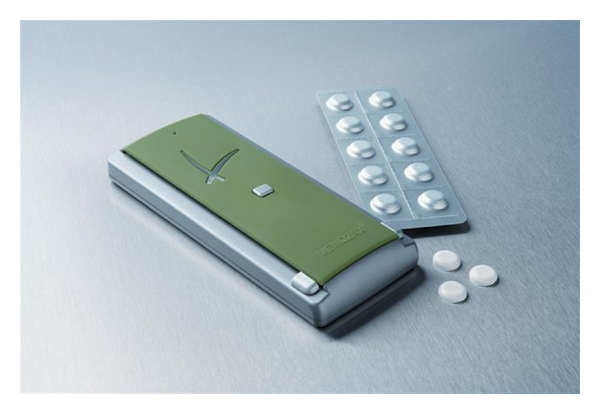
The compliance device (Memozax) used in the trial.

A flowchart of subjects disposition is presented in Figure 1. The subject demographic values, smoking history, and allergy disease history at baseline are summarised in Table 1. There were no major baseline differences between the two groups. It is to note that 73% of the enrolled patients suffered from moderate/severe allergic rhinoconjunctivitis. A total of 50 patients (25 in both groups) also reported asthma (19% of FAS population). Monosensitive patients (subjects with SPT positive only for grass extracts) were 68 (26%) and multisensitive patients (subjects with also at least one positive SPT toward nongrass allergens) were 193 (74%).

### 4.1. Primary Endpoint: Compliance

The overall mean compliance rate was 91.3% (median 97%) for the subjects with complete compliance data from visit 3 to visit 5. The primary endpoint in this trial was a comparison of the degree of compliance in the two groups (Memozax and non-Memozax). For this purpose compliance was categorised as *excellent *(≥90%) or *less excellent* (<90%). The proportion of subjects with *excellent *compliance in the Memozax group was similar (79%) to that in the non-Memozax group (78%) (Table 2). The difference was not statistically significant (*P* = 0.5).

### 4.2. Secondary Endpoints: Clinical Efficacy, Tolerability, and Safety

Other efficacy assessment was to evaluate after 48 weeks of treatment with Grazax tablets the impact on quality of life, symptom score, and patient's acceptance in comparison with previous pollen seasons. This was evaluated, globally, through a 10 cm VAS scale (0 = big improvement, 5 = not improvement, and 10 = worsening of symptoms) which was performed by the patient. At visit 5 the mean VAS score was 2.4 ± 1.8 showing a general clinical improvement. The percentage of patients with a VAS score = or >5 (no-difference/worsening in comparison with the previous season) was 19%. Therefore 81% of patients reported a clinical improvement of symptoms after treatment with Grazax in comparison with the previous year. Clinical efficacy was comparable both in monosensitized patients (68 out of 261: 26%) and in multisensitized subjects (193 out of 261: 74%). Investigator Global Clinical evaluation of 48-week treatment with Grazax was good/very good: 85%; sufficient: 9%; not good: 6%. The percentage of subjects who reported adverse events (AEs) in each of the two groups was almost similar, 14% in the Memozax group and 11% in the non-Memozax group. A total of 63 AEs (78%) reported were judged as probably or possibly related to immunotherapy by the investigator, while 14 reported AEs were judged as unlikely or not related to allergy tablet treatment. For 2 AEs, the investigator did not report the causality. The majority of AEs (71 out of 79: 90%) were either mild or moderate, with only 4% (absolute number: 3) of AE reported as severe AE (mouth itching). No serious adverse events were observed in the study.

## 5. Discussion

Allergen-specific immunotherapy is the only causative treatment of several allergy diseases. The main feature of this therapeutic approach is its capacity to modify the natural history of the disease, reducing the development of asthma and new sensitizations after 3-4 years of treatment [17]. Adequate compliance to allergen-specific SLIT is, however, mandatory in order to obtain these results.

This study trial investigated the compliance of 48-week grass allergy tablet treatment in two groups of subjects, one issued with the Memozax compliance device and the other not issued with the device. Overall compliance with grass AIT was high (>90%). The compliance rate in the Memozax group was slightly higher (91.7%) than that in the non-Memozax group (90.3%), but the difference was not statistically significant. A total of 79% of the patients in the Memozax group who completed the trial had a compliance >90%. In the group without Memozax this percentage was 78%. In the global evaluation, a total of 81% of patients reported an improvement of symptoms after treatment with grass AIT, evaluated through a 10 cm VAS in comparison with the previous season. Investigators evaluated the efficacy of treatment as good or very good in 85% of patients. In this trial clinical efficacy was of similar extent both in monosensitive and polysensitive patients. This is a strong indication that treatment with grass is effective in relieving these symptoms, and it is in line with results from previous grass AIT trials. The safety profile seen in this trial reflects the overall good tolerance to grass allergen tablet treatment [18]. In this study no serious adverse events or deaths were observed. In conclusion compliance to the treatment with grass AIT administered every day is in general high. In this specific clinical setting, the use of electronic devices is not associated with a greater compliance. In addition this trial supports the safety, tolerability, and efficacy profile observed in previous trials of this grass allergy immunotherapy tablet in grass allergic patients.

## Figures and Tables

**Table 1:** Patients demographic characteristics at baseline.

Treatment Group	Grazax+ Memozax	Grazax− Memozax
Number of subjects	139	122
Age (years) #		
*N *	139	122
Mean (SD)	32 (9)	33 (10)
Median	32.0	33.0
Min-Max	19–60	18–63
Sex		
*N *	139	22
Men	75 (54%)	74 (60%)
Women	64 (46%)	48 (40%)
Allergic rhinitis		
Mild	37 (26%)	33 (27%)
Moderate/severe	102 (74%)	89 (73%)
Monosensitive subjects	32 (23%)	36 (29%)
Polysensitive subjects	107 (77%)	86 (71%)
Asthma (Gina class: I–III)		
*N *	25 (18%)	25 (20%)

*N*: Number of subjects; (%): Percent of subjects.

**Table 2:** Excellent compliance versus less excellent compliance.

Treatment Group	Grazax+		Grazax−	
	Memozax		Memozax	
	*N*	(%)	*N*	(%)
Number of subjects	139		122	
Primary analysis no.				
*N *	113		99	
Excellent compliance (≥90%)	90	(79%)	78	(78%)
Less excellent compliance (<90%)	23	(21%)	21	(22%)

*N*: Number of subjects; (%): Percent subjects.
